# Sugar ring alignment and dynamics underline cytarabine and gemcitabine inhibition on Pol η catalyzed DNA synthesis

**DOI:** 10.1016/j.jbc.2024.107361

**Published:** 2024-05-10

**Authors:** Caleb Chang, Grace Zhou, Christie Lee Luo, Sarah Eleraky, Madeline Moradi, Yang Gao

**Affiliations:** Department of Biosciences, Rice University, Houston, Texas, USA

**Keywords:** nucleoside analogue drugs, DNA polymerase, time-resolved X-ray crystallography, sugar pucker, DNA extension, primer alignment

## Abstract

Nucleoside analogue drugs are pervasively used as antiviral and chemotherapy agents. Cytarabine and gemcitabine are anti-cancer nucleoside analogue drugs that contain C2′ modifications on the sugar ring. Despite carrying all the required functional groups for DNA synthesis, these two compounds inhibit DNA extension once incorporated into DNA. It remains unclear how the C2′ modifications on cytarabine and gemcitabine affect the polymerase active site during substrate binding and DNA extension. Using steady-state kinetics, static and time-resolved X-ray crystallography with DNA polymerase η (Pol η) as a model system, we showed that the sugar ring C2′ chemical groups on cytarabine and gemcitabine snugly fit within the Pol η active site without occluding the steric gate. During DNA extension, Pol η can extend past gemcitabine but with much lower efficiency past cytarabine. The Pol η crystal structures show that the -OH modification in the β direction on cytarabine locks the sugar ring in an unfavorable C2′-endo geometry for product formation. On the other hand, the addition of fluorine atoms on gemcitabine alters the proper conformational transition of the sugar ring for DNA synthesis. Our study illustrates mechanistic insights into chemotherapeutic drug inhibition and resistance and guides future optimization of nucleoside analogue drugs.

Nucleoside analogue drugs are widely used as antiviral and anticancer chemotherapeutic agents (1, 2). Many of these nucleotide-mimicking compounds hinder DNA or RNA synthesis by carrying modifications such as ring-opening bonds, heteroatoms, L/D stereochemistry, and bulky chemical groups on the nitrogenous base, phosphate region, or the sugar ring. A large class of potent nucleoside analogue drugs lacks a functional 3′-OH on the sugar ring (3). After a DNA polymerase incorporates these drugs onto an elongating DNA chain, the nucleophilic attack for the next round of DNA synthesis cannot proceed without the 3′-OH group. However, there exist nucleoside analogue drugs that inhibit DNA extension despite containing a functional 3′-OH group such as vidarabine, cytarabine (araC), and gemcitabine (gemC) (4, 5, 6). AraC, used in many leukemia treatments (7, 8, 9), is a lethal cytosine analogue that contains a hydroxyl (-OH) group on the 2′ carbon pointing in the β direction (Fig. 1*A*). Commonly prescribed for solid tumors, gemC is a chemotherapy cytosine analogue that contains two fluorine (-F) atoms on the 2′ carbon (Fig. 1*A*). Despite containing functional 3′-OH groups, these two compounds are proposed to act as chain terminators for DNA polymerases by inhibiting DNA extension (10, 11, 12, 13, 14, 15). Previous studies indicated that Pol β and Pol λ can incorporate araCTP and gemCTP, with the latter being less efficient (16, 17). Moreover, the Pol λ structure with gemCTP suggested that the ribose sugar of gemCTP shifted by 0.9 Å away from the primer end, explaining why gemCTP is less favored than araCTP. In contrast, Pol α unfavorably inserts araCTP and gemCTP and inefficiently bypasses gemC or araC on the template (15). Similarly, gemCTP incorporation and template bypass is not as efficient by Pol γ (14). In 2018, Rechkoblit and colleagues showed that Pol η can extend past araC-terminated DNA and captured a structure with the araC primer at the DNA extension step (18). In the structure, the Me^2+^_A_ is weakly bound and the araC primer 3′-OH lies too far to initiate a nucleophilic attack. Despite such efforts, there lacks a detailed and comprehensive study that investigates both incorporation and extension with kinetic and structural approaches for these analogue drugs. Additionally, drug resistance is prevalent among patients who are prescribed long-term with araC (19, 20) and gemC (21, 22), and how cellular DNA repair enzymes tolerate and bypass these abnormal nucleosides remains largely unclear.

**Figure 1:**
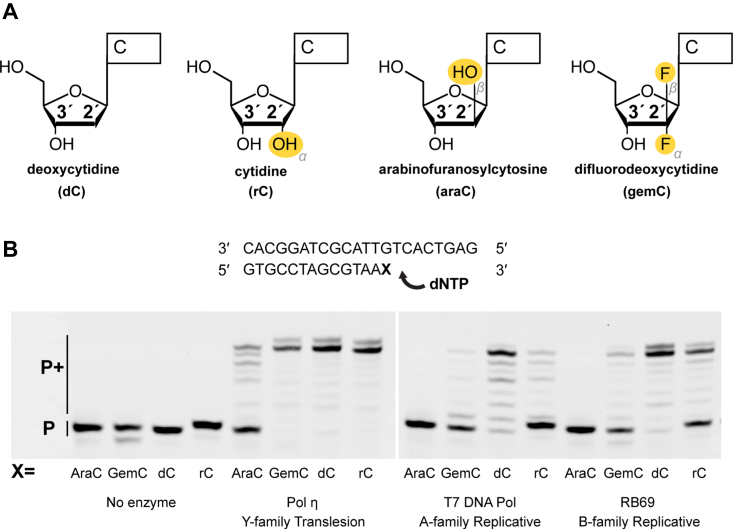
**Biochemical characterizations of Pol η and replicative polymerases bypassing primer ends with different dC analogues.***A*, chemical structure of nucleoside analogue drugs araC and gemC in comparison to dC and rC. *B*, primer extension assay from araC-, gemC-, rC-, and dC-terminated DNA by Pol η, T7 DNA Pol, and RB69 polymerase. The most bottom bands are denoted by “P,” which represents unextended primer DNA, while the bands above the P (P+) represent primer DNA following nucleotide addition.

Polymerase η (Pol η) is a Y-family translesion polymerase expressed to bypass bulky DNA lesions (23, 24). One of the major DNA lesion substrates for Pol η is the pyrimidine thymine dimer induced by UV-light overexposure (23, 24). Patients who carry mutations on the Pol η gene develop UV sensitivity or a predisposition for xeroderma pigmentosum skin cancer (25, 26). Conversely, Pol η overexpression has been linked to the development of chemoresistance and reduced cisplatin sensitivity in several cancers, including lung, ovarian, and bladder cancers (27). Moreover, UV-damage-mediated cellular arrest can be prevented through the recruitment of Pol η in the ATR pathway (28). Knocking out Pol η is synthetically lethal to ATR-deficient cells (29). Thus, Pol η has been proposed (30) and pursued (31, 32, 33) as a promising target for combined cancer therapy.

The DNA synthesis reaction by Pol η has been extensively studied. Previous time-resolved crystallographic studies revealed the DNA synthesis process in Pol η (34, 35, 36, 37, 38, 39): first, DNA binds within the active site followed by the incoming nucleotide and the B site metal ion (Me^2+^_B_); afterward, the A site metal ion (Me^2+^_A_) binds, which leads to the alignment of the primer terminus; then product formation occurs in conjunction with C site metal ion binding between the P_α_ and P_β_ oxygens of the incoming nucleotide, as well as sugar pucker transition from C2′-endo to C3′-endo of the primer terminus; after pyrophosphate leaves the active site, the inserted nucleotide is translocated to the primer terminus region for the next DNA synthesis reaction. During DNA synthesis, polymerase efficiency and substrate discrimination are correlated with the dynamics and conformation of the primer terminal nucleotide and its 3′-OH group (39, 40). An additional -OH group on the 2′ carbon in the α direction, such as with ribonucleotides (Fig. 1*A*) at the primer terminus encourages substrate alignment and locks the sugar pucker into a C3′-endo conformation. The C3′-endo conformation of the primer terminus leads to increased polymerase efficiency but decreased substrate discrimination (41). These previous mechanistic studies on Pol η not only advance the understanding of the DNA synthesis reaction process but also highlight the essential correlation among nucleoside sugar modification, primer-end dynamics, and Pol η DNA synthesis.

It has been reported Pol η broadly participates during DNA replication at stalled replication forks (42) and that knocking out the Pol η gene sensitizes cancer cell lines to araC and gemC. Cellular DNA extension assays show that Pol η can extend past araC and gemC-terminated DNA, conferring chemoresistance (10). Despite existing kinetic and structural studies on araC and gemC on DNA polymerases, the relationship between the primer terminus conformation, sugar modification, and inhibition of DNA extension is not well defined, and the dynamics of the araC primer was unexplored. Moreover, how Pol η tolerates araC within its active site during incorporation was also not captured, and how Pol η interacts with and extends past gemcitabine has not been investigated. With araC and gemC differing only on the 2′ carbon compared to deoxynucleotides and ribonucleotides (Fig. 1*A*), we hypothesize that the 2′-modifications on araC and gemC may affect DNA extension by altering sugar pucker dynamics and primer alignment. Here, we report that Pol η can incorporate araCTP and gemCTP, the active triphosphate forms of the drugs, into DNA with slightly less efficiency compared to dCTP. Both araCTP and gemCTP bind within the Pol η active site with similar base pair geometries and minimal steric interactions compared to dCTP. Once incorporated and translocated to the primer terminus, the static and *in crystallo* structural snapshots of Pol η show that araC misaligns the primer and locks the sugar ring in a C2′-endo conformation unfavorable for nucleophilic attack. In contrast, the gemC primer terminus only alters the proper conformational transition of the sugar ring but still allows the active site to properly assemble, which correlates with its weaker inhibition on DNA extension compared to araC. Our results illustrate the mechanism of inhibition of araC and gemC and also the structural basis for resistance conferred by Pol η.

## Results

### Biochemical studies of Pol η inserting and extending past araCTP and gemCTP

We first tested DNA extension past araC or gemC with model replicative polymerases, the A-family T7 DNA polymerase (T7 Pol), which is homologous to human polymerase γ, and the B-family RB69 polymerase (RB69), which is homologous to human polymerase α, δ, and ε. We also tested a C-terminal truncated Pol η, which lacks the disordered C-terminal region for protein-protein interactions and has a similar level of polymerase activity as the full-length enzyme (43). The exonuclease knockout variants of both T7 Pol and RB69 were unable to extend DNA past araC and had limited abilities in extending beyond gemC (Fig. 1*B*), similarly as reported for polymerase α and γ (14, 15). In contrast, Pol η could efficiently extend beyond araC and gemC, consistent with the cellular bypassing assays and previous biochemical results (10). The data confirmed that araC and gemC can inhibit DNA extension by replicative polymerases, whereas Pol η can bypass araC or gemC to promote drug resistance.

**Figure 2:**
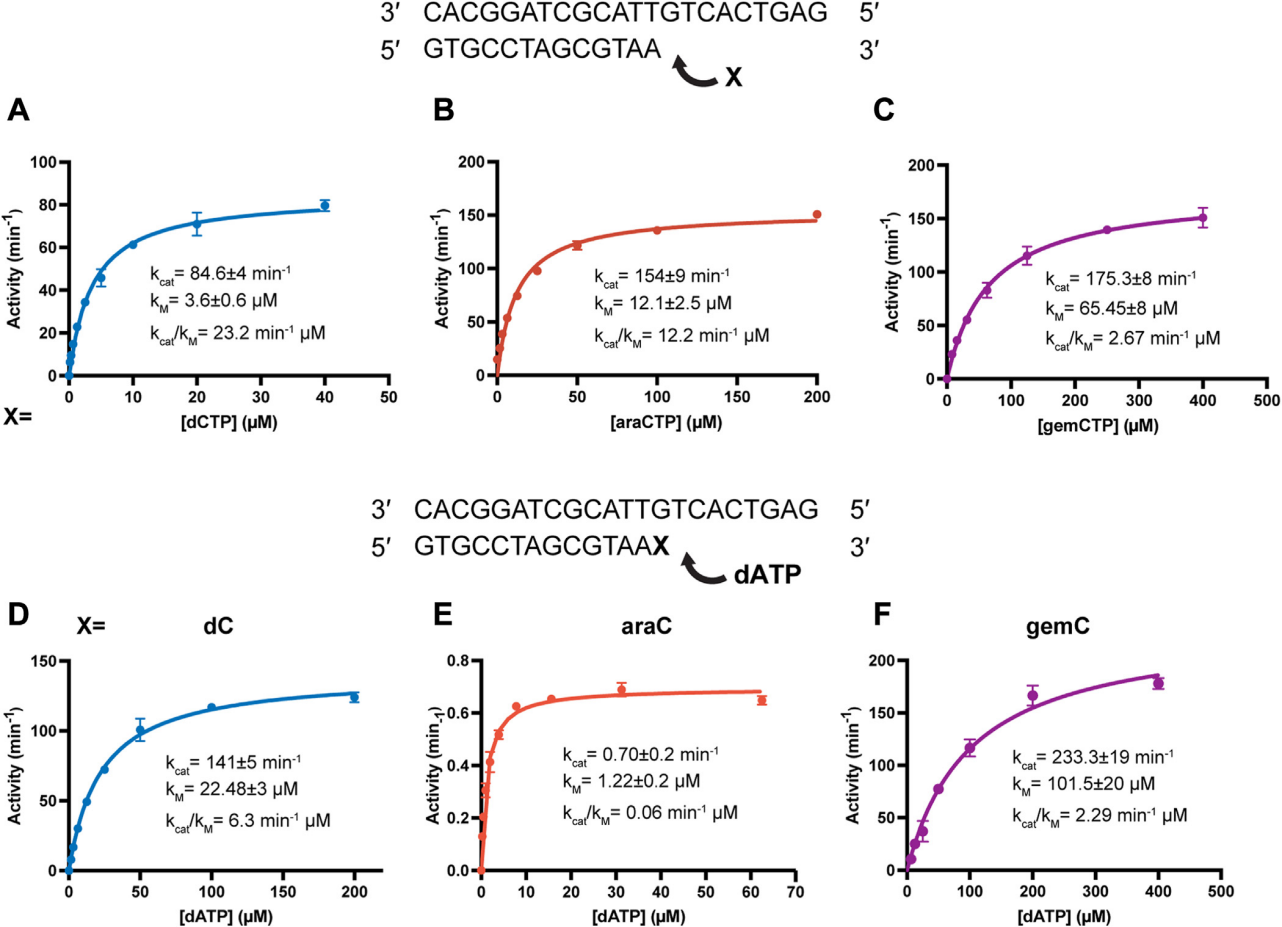
**Steady-state kinetics of Pol η during incorporation and extension of deoxycytidine, cytarabine, and gemcitabine.***A–C*, steady-state kinetics of single-nucleotide incorporation of dCTP (*A*), araCTP (*B*), and gemCTP (*C*) by Pol η. The catalytic efficiency (k_cat_/k_M_) only dropped by 2-fold for araCTP but 9-fold for gemCTP. *D–F*, steady-state kinetics of extension from dC (*D*), araC (*E*), and gemC (*F*) by Pol η. The catalytic efficiency (k_cat_/k_M_) dropped by 100-fold for araCTP but 2-fold for gemCTP. In all panels, each data point represents the mean of triplicate measurements for the polymerase incorporation rate while the errors bars represent the standard deviation.

We then kinetically characterized the incorporation of araCTP and gemCTP by Pol η. Single-nucleotide incorporation assays indicated that Pol η incorporated araCTP (K_cat_/K_m_ of ∼12 μM^−1^ min^−1^) efficiently, as compared to dCTP (K_cat_/K_m_ of ∼23 μM^−1^ min^−1^) (Fig. 2, *A* and *B*). Similarly, Pol η could incorporate gemCTP as well with a K_cat_/K_m_ of ∼2.7 μM^−1^ min^−1^. Together, incorporating dCTP was only 2-fold more efficient than araCTP and 9-fold more efficient than gemCTP even though both gemCTP and the ribonucleotide cytidine triphosphate (rCTP) contain a chemical group on the C2′ carbon that points in the α direction (Fig. 2, *A* and *B* and Table 1).

**Table 1:** Steady-state kinetics of dCTP, araCTP, and gemCTP incorporation by WT Pol η

Primer terminus	dA	dA	dA	dA
Incoming dNTP	dCTP	araCTP	gemCTP	rCTP
K_M_ (μM)	3.64 ± 0.6	12.1 ± 2.5	65.40 ± 8	132.5 ± 15
K_cat_ (min^−1^)	84.63 ± 4	153.6 ± 9	175.30 ± 8	1.227 ± 0.05
K_cat_/K_M_ (min^−1^ μM)	23.20	12.20	2.67	0.009
Ratio		1.90	8.69	2578

**Figure 3:**
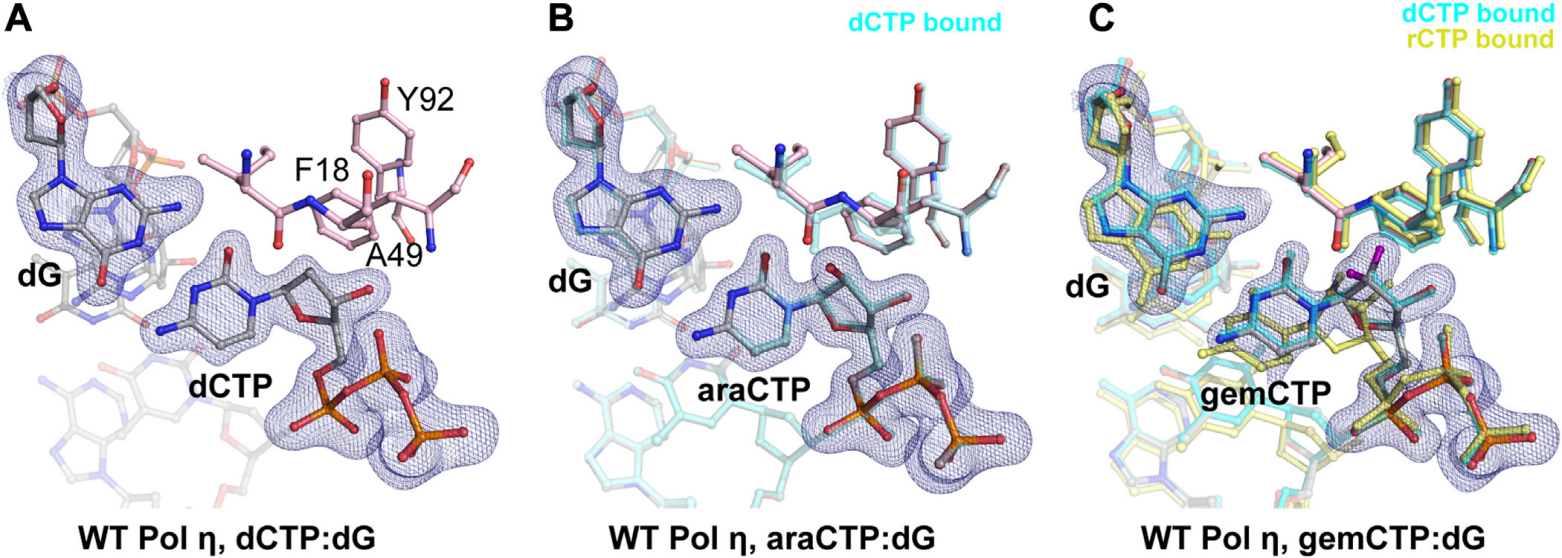
**Insertion complexes of Pol η with dCTP analogues.***A–C*, active site structure of Pol η with dCTP as the incoming nucleotide (*A*), araCTP as the incoming nucleotide (*B*), and gemCTP as the incoming nucleotide (*C*). The dCTP is shown as cyan sticks and overlaid with araCTP (*B*) and gemCTP (*C*). The rCTP is shown as yellow sticks and overlaid with the gemCTP (*C*). The fluorine atoms on gemCTP are colored in purple. The Fo-Fc map for dCTP (*A*), araCTP (*B*), and gemCTP (*C*) were contoured at three σ. Base-pairing geometry of araCTP and gemCTP are nearly identical to that for dCTP.

During the extension step following araCTP and gemCTP insertion, Pol η extended from araC 100-fold less efficiently with a K_cat_/K_m_ of 0.06 μM^−1^ min^−1^ as opposed to 6.3 μM^−1^ min^−1^ for dCTP. The assays revealed that extending from gemC was only 3-fold less efficient *versus* from dC (Fig. 3 and Table 2). Our kinetic results were consistent with the dNTP extension assay, which showed Pol η, T7 DNA polymerase, and RB69 polymerase converted more product from gemC-terminal DNA than araC-terminal DNA (Fig. 1*B*).

**Table 2:** Steady-state kinetics of dC, araC, and gemC extension by WT Pol η

Primer terminus	dC	araC	gemC
Incoming dNTP	dATP	dATP	dATP
K_M_ (μM)	22.48 ± 3	1.22 ± 0.2	101.50 ± 20
K_cat_ (min^−1^)	141.10 ± 5	0.70 ± 0.2	233.30 ± 19
K_cat_/K_M_ (min^−1^ μM)	6.30	0.06	2.29
Ratio		105	2.75

### Pol η structures of nucleoside drug insertion

To investigate how the 2′-modifications on araCTP and gemCTP affect their incorporation, we produced crystal structures of Pol η with araCTP and gemCTP within the insertion site. Interestingly, Pol η′s active site tolerated the 2′-OH modification on araC quite adequately. The sugar pucker of araCTP existed in a C3′-endo conformation with the 2′-OH pointing 60˚ away from Phe18, a crucial residue of the steric gate that prevents the binding and incorporation of ribonucleotides (44). The insertion site around the backbone of Ala49 expanded by only 0.2 Å to accommodate the 2′-OH. Despite containing an additional 2′-OH, araCTP was inserted nearly identical as a coplanar classic Watson-Crick dCTP:dG basepair (Fig. 3), and the dT primer terminus was aligned to the α-phosphate of araCTP ready for nucleophilic attack.

Similar to araCTP, the additional 2′-F atoms on gemC did not significantly alter the active site. Besides minimal 0.2 Å movement around the Ala49 backbone, Phe18 and Tyr92 moved by 0.3 Å to accommodate the additional F atoms. Like dCTP and araCTP, gemCTP is basepaired in a C3′-endo conformation and coplanar to the template dG with 1 F atom pointing towards the backbone of Ala49 and the other F atom pointing towards the Phe18 and Tyr92 steric gate. Aside from rCTP also binding in a C3′-endo conformation (Fig. S1), gemCTP base-pairing geometry was distinctive from that of rCTP, which also contains a C2′ modification that points towards the Pol η steric gate. GemCTP’s C2′ carbon only moved 0.3 Å away from the steric gate, whereas the C2′-OH group on rCTP distorted the ribonucleotide to move 2.2 Å. As a consequence, this caused rCTP to basepair with a propeller twist geometry without clashing with the steric gate (Figs. 2*C* and 3*C*, S1). In summary, our crystal structures revealed that araCTP and gemCTP favorably bind within the active site for incorporation without significantly perturbing the active site.

### AraC at the DNA primer end alters the sugar pucker conformation

**Figure 4:**
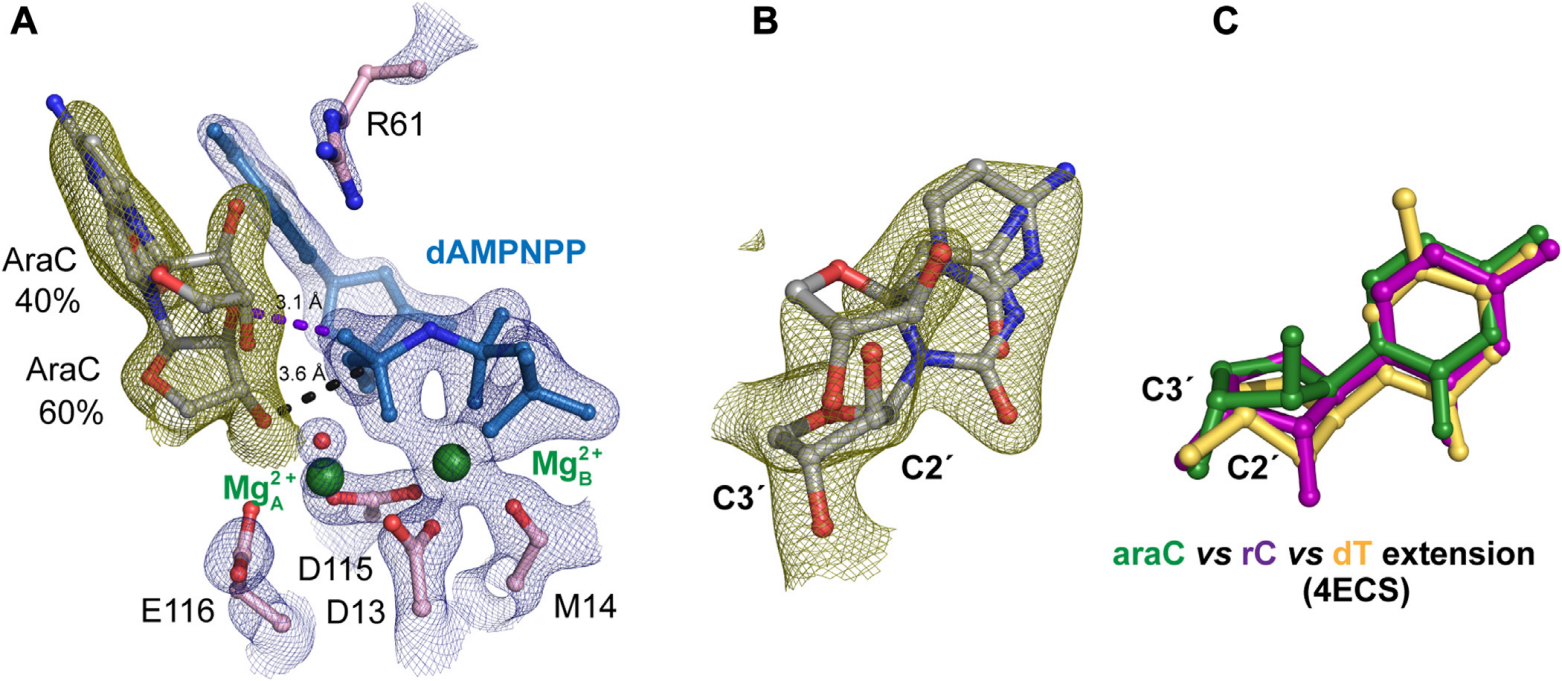
**Ternary complex structure of Pol η with an araC at the primer terminus.***A*, active site of the araC extension complex with Mg^2+^, in which the araC primer terminus is partially misaligned with the α-phosphate. *B*, zoom-in view of araC at the primer terminus revealed both aligned and misaligned conformations of the araC during extension. *C*, structural overlay of araC (*green*), rC (*purple*), and dT (product state) (PDB ID 4ECS) (*yellow*) at the primer terminus. The differences in sugar pucker geometry are shown. *A* and *B*, The 2F_o_-F_c_ map for Me^2+^_A_ and Me^2+^_B_, dAMPNPP, water (*red sphere*), and catalytic residues (*blue*) was contoured at two σ. The F_o_-F_c_ omit map for the primer terminus (*green*) was contoured at 2.5 σ.

To investigate how araC affects the polymerase active site during DNA extension, we prepared Pol η crystals complexed with araC-terminated DNA. Within the active site, the nonhydrolyzable nucleotide analogue dAMPNPP binds in a geometry similar to dATP against the template dT with Mg^2+^ clearly bound at the Me^2+^_B_ site (Figs. 4, S2, and S3). However, 40% of the primer terminus araC was misaligned in the up-shifted conformation. The up-shifted AraC base was stabilized by the sidechain of Arg61, which stayed 3.6 Å above the incoming nucleotide phosphate to interact with the upper-shifted primer. At the same time, 60% of the primer terminus remained in a down conformation. The down conformation of the araC primer laid 168˚ and 3.6 Å from the incoming nucleotide α-phosphate compared to a dT primer end, which exists 172˚ and 3.3 Å away. In the study by Rechkoblit and colleagues, the araC primer was captured in a single down conformation existing 160° and 4.3 Å from the target phosphate (Fig. S2). Within our structure, the sugar pucker for both down and up-shifted conformations existed in a C2′-endo conformation due to the β-2′-OH modification. Modeling the sugar in a C3′-endo resulted in steric clashes between the 2′-OH and sugar ring of the incoming nucleotide. In addition, very weak electron density existed in the vicinity of the Me^2+^_A_ binding site, and the Mg^2+^_A_ could only be modeled with 30% occupancy. Such reduced Mg^2+^ affinity for the Me^2+^_A_ site was likely due to the misalignment and the altered conformation of the araC primer (Fig. 4).

### AraC at the DNA primer end locks in a C2′-endo sugar pucker

**Figure 5:**
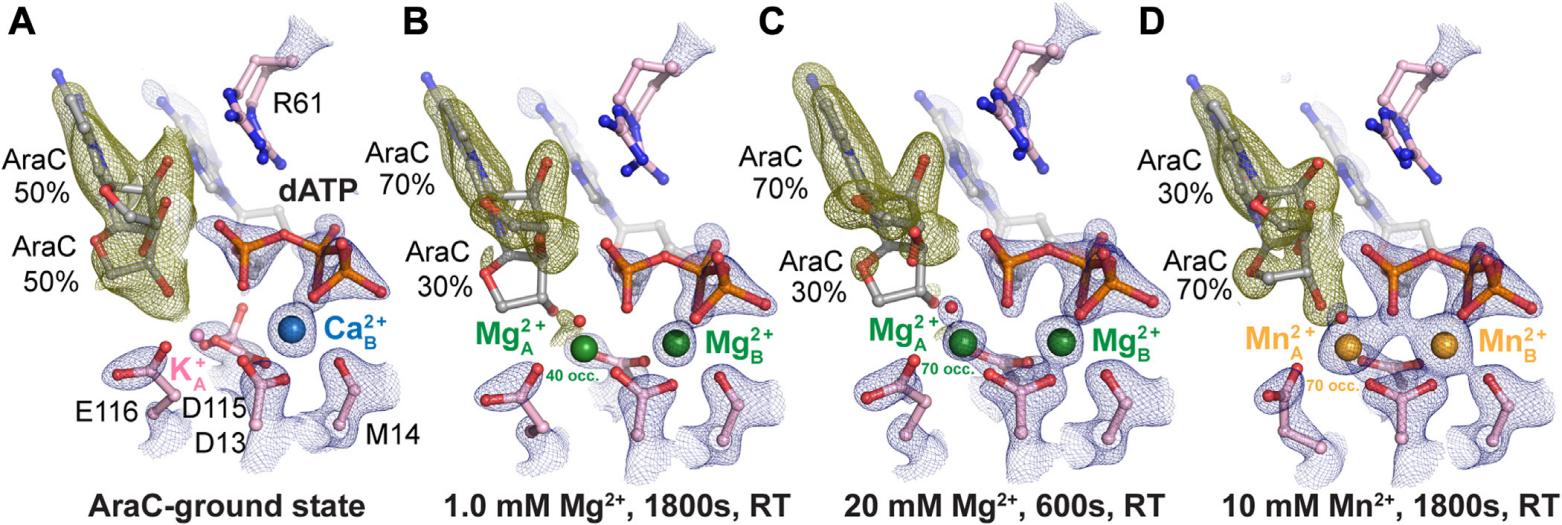
***In crystallo* visualization of araC extension complex with Mg^2+^ or Mn^2+^.***A*, active site structure of the Pol η ground state during araC extension. A single Ca^2+^ (blue) was shown to bind within the active site. *B–D*, active site structures of Pol η following *in crystallo* soaking at different conditions: 1 mM Mg^2+^ soaking for 1800s (*B*), 20 mM Mg^2+^ soaking for 600s (*C*), and 10 mM Mn^2+^ soaking for 1800s (*D*). The 2F_o_-F_c_ map for the Me^2+^_A_ and Me^2+^_B_, dAMPNPP, water (*red sphere*), and catalytic residues (*blue*) was contoured at two σ. The F_o_-F_c_ omit map for the primer terminus (*green*) was contoured at 2.5 σ.

To further illustrate how the 2′-OH modification affects the sugar pucker conformation and primer end dynamics, we made Pol η ternary complex crystals with an araC primer end, incoming dATP, and Ca^2+^, for *in crystallo* soaking experiments with Mg^2+^ or Mn^2+^ (Fig. S4). With Ca^2+^, 50% of the araC primer was in the down conformation. The Me^2+^_B_ site was fully saturated with Ca^2+^, but the Me^2+^_A_ site was partially occupied with K^+^ (Fig. 5*A*). The down conformation of the araC primer existed 3.5 Å away from the target α-phosphate. After the crystals were soaked in 1 mM Mg^2+^ for 30 min, 70% of the araC primer remained in the up-shifted conformation (Fig. 5*B*), leaving the octahedral shell of the Me^2+^_A_ incomplete. Correspondingly, reduced electron density was detected for the Me^2+^_A_ site compared to the Me^2+^_B_ site. We then attempted to soak the Pol η crystals in a higher concentration of Mg^2+^ to investigate whether Mg^2+^ binding at the A-site was hindered due to the 2′-modification. Our structure suggested that Mg^2+^ could still bind to the A-site as indicated by the increased electron density (Fig. 5*C*). However, the primer end remained misaligned and no reaction product was detected. Subsequently, we soaked the crystals in 10 mM Mn^2+^, which has been shown to promote primer end alignment and misincorporation (39). With Mn^2+^, 30% of the araC primer existed in the up-shifted conformation while both Me^2+^_A_ and Me^2+^_B_ sites showed strong electron density (Fig. 5*D*). Even though Mn^2+^ saturated both Me^2+^_A_ and Me^2+^_B_ sites, product density was still not detected after 30 min soaking. Interestingly, in all of these Mg^2+^ or Mn^2+^ soaked crystals, the sugar ring of the araC primer existed in a C2′-endo conformation. Thus, the inhibition of product formation was likely due to primer misalignment and increased rigidity of the sugar pucker rather than Me^2+^_A_ binding. Moreover, the relatively rigid crystalline environment reduced the Brownian motion of the primer end, which may have further restricted DNA extension.

### The sugar ring is destabilized during GemC extension

**Figure 6:**
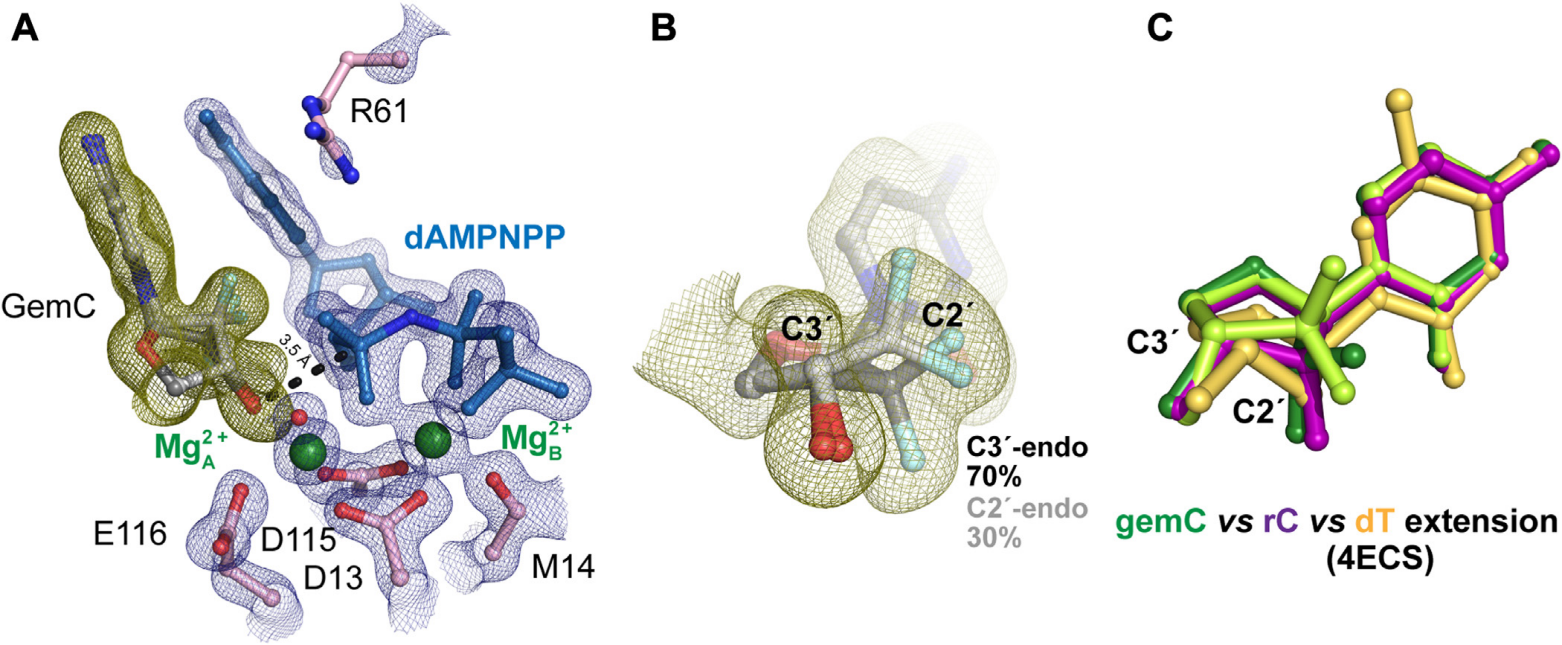
**Ternary complex structure of Pol η with gemC at the primer terminus.***A*, active site structure of gemC extension complex with Mg^2+^. 30% of the gemC primer terminus sugar pucker exists as a C2′-endo while 70% of the gemC primer terminus sugar pucker exists as a C3′-endo. The gemC primer terminus lies 3.5 Å from the target α-phosphate. *B*, zoom-in view of gemC at the primer terminus. Electron density for the gemC primer terminus sugar ring indicates a mixture of sugar ring geometry. *C*, structural overlay of gemC (*green*), rC (*purple*), and dT (product state) (PDB ID 4ECS) (*yellow*) at the primer terminus. The alignment indicated the differences in sugar pucker geometry. *A* and *B*, the 2F_o_-F_c_ map for Me^2+^_A_ and Me^2+^_B_, dAMPNPP, water (*red sphere*), and catalytic residues (*blue*) was contoured at two σ. The F_o_-F_c_ omit map for the primer terminus (*green*) was contoured at 2.5 σ.

To investigate why DNA extension is not as inhibited by gemC, we created Pol η crystals in complex with gemC-terminated DNA. When gemC translocated to the primer terminus, the incoming nucleotide dAMPNPP inserted in a Watson-Crick basepair conformation (Fig. S5). The electron density showed that all of the gemC primer terminus was aligned and existed 3.5 Å away from the α-phosphate (Fig. 6*A*). However, 30% existed in a C2′-endo conformation while 70% of the sugar ring existed as a C3′-endo conformation (Fig. 6*B*). In contrast to the araC extension structure, the Me^2+^_A_ site was fully saturated. Interestingly, Arg61 occupied the same location as in the araC structure but remained too far from the F atom in the α direction for hydrogen bonding (Fig. S6). Although the 2 F atoms on gemC pointed towards the nitrogenous base of the incoming nucleotide, the position of dAMPNPP changed by only 1 Å (Figs. 6*C*, S5). Aside from 30% of the gemC primer terminus existing in a C2′-endo sugar pucker and slightly too far from the incoming nucleotide, the active site looked nearly optimal for nucleophilic attack, consistent with the biochemical results.

## Discussion

Numerous past studies have sought to comprehend the mechanistic basis for the inhibition of various DNA polymerases, including Pol α, β, γ, and λ, by araC and gemC using kinetics or structural methods, but rarely both. Some studies have been limited to understanding the presence of the analogue drug in one process, including the insertion step, extension step, or template bypass. Given Pol η′s extensive role during DNA replication (42) and major role in bypassing araC and gemC (10), our comprehensive analysis of Pol η during both incorporation and extension from araC and gemC reveals critical details on AraC's strong inhibition mechanism and gemC's weaker inhibition.

In order to act as a potent chain terminator, a nucleoside analogue must be well tolerated within the polymerase active site and efficiently incorporated to the DNA end. Many DNA polymerases contain a steric gate comprised of aromatic residues to prevent the stable binding of ribonucleotides within the active site (44, 45, 46, 47, 48, 49, 50). The presence of an -OH group on the C2′ carbon sugar ring in the α direction sterically clashes with the steric gate. When rCTP was inserted within Pol η, the additional 2′-OH group in the α direction sterically clashed with Tyr92 and Phe18 of the steric gate, forcing the ribonucleotide to basepair in a propeller twist geometry (Fig. S1). Here, we showed that the -OH group in the β direction on cytarabine and the -F groups on gemcitabine minimally occluded the steric gate of Pol η, thus allowing stable substrate binding. Structural distortion of rCTP *versus* araCTP within the active site highlights the importance of the direction of the -OH modification for efficient insertion. On the other hand, the F atom on gemcitabine that points toward the steric gate did not significantly distort base pairing (Fig. 3*C*). The reduced steric clash of gemCTP in contrast to rCTP was likely due to the shorter length of the C-F bond and/or the more electronegative nature of F compared to OH (51, 52). In contrast, gemCTP binds weakly and sterically clashes within the active sites of X-family Pol λ and β. Notably, the steric gate in Pol β is mainly comprised of amino acid backbones, whereas in Pol λ, both the protein backbone and the Y505 sidechain constitute the steric gate (14, 16, 17, 53). Minimal occlusion of the Pol η active site by the modifications of araC and gemC explains why they can be efficiently incorporated into DNA to later inhibit DNA extension.

During nucleophilic attack and product formation, the primer terminus sugar pucker needs to transit to a C3′-endo conformation (41). Modifications on the sugar ring can affect the sugar pucker conformation and sugar pucker transition, thereby inhibiting nucleophilic attack and product formation. After araC was inserted and translocated to the primer terminus, the 2′-OH modification promoted the primer terminus to misalign with the substrate α-phosphate and locked the sugar ring in a C2′-endo pucker. We speculate that the altered sugar pucker conformation and dynamics of the primer termini underline the inhibition effect of araC. Regarding gemcitabine, although it was previously assumed that the two dipoles from the F atoms on gemcitabine would cancel out, 70% of the sugar ring existed in a C3′-endo sugar pucker while 30% existed in a C2′-endo pucker. This dynamic conformation is corroborated by NMR studies (54) and contrasts with native deoxynucleotide extension, where all of the DNA sugar ring exists in a single conformation (34, 35, 39). Other than altered sugar ring dynamics, Pol η could efficiently bypass gemC, with its active site moderately distorted by gemC at the primer terminus. Our findings corroborate Pol η′s role in conferring drug resistance (10). At the same time, we acknowledge that Pol η is a Y-family polymerase specialized in translesion DNA synthesis, which may have different properties from replicative polymerases in bypassing araC and gemC. Notably, RB69 and T7 DNA polymerases, serving as analogues to human replicative polymerases, exhibited markedly greater inhibition during araC and gemC extension compared to Pol η (Fig. 1*B*). Similarly, previous studies have shown that replicative Pol ε is inhibited during RNA extension (55), further underscoring the potential role of translesion polymerases in bypassing these nucleoside analogue drugs. However, the precise impact of the altered sugar pucker conformation and dynamics of gemC and ribonucleotide at the primer terminus on replicative polymerase extension warrants further investigation.

Sugar ring modifications are common in existing nucleoside analogue drugs. For instance, vidarabine, identical to cytarabine except for having an adenine instead of a cytidine base, inhibits DNA synthesis against herpes simplex virus, poxvirus, and vesicular stomatitis virus infections (1). In addition, clofarabine, which contains a single -F atom in the β direction on the C2′ carbon, is a potent leukemia nucleoside analogue drug that inhibits reductases (56), HIV-RT (56), and polymerases α and ε (57, 58, 59, 60). Computational simulations have suggested that a -F *versus* an -OH modification on the C2′ carbon can further lock the nucleoside analogue in a C2′-endo sugar pucker (61). We speculate that sugar ring modifications on vidarabine and clofarabine may inhibit DNA extension through a similar mechanism to that of cytarabine. Similarly, entecavir, which contains a double bond in place of an oxygen atom on the sugar ring, strongly inhibits the hepatitis B viral DNA polymerase (62). Remdesivir is a potent Sars-CoV-2 cytidine analogue that contains a cyano group on the C1′ position (63). Although Remdesivir can be efficiently incorporated, the cyano group sterically clashes with a serine residue in the translocation tunnel after three nucleotide additions, restricting further nucleotide incorporations (63, 64). These previous studies highlight the significance of sugar ring modifications in affecting the polymerase reaction, especially during the extension step following drug incorporation. Although nucleoside analogue drugs with single modifications have been extensively investigated, further exploration involving combining modifications at different positions, along with base modifications, may lead to the design of more potent novel nucleoside analogue drugs for cancer and virus treatment.

## Experimental procedures

### Protein expression and purification

Wild-type human polymerase η (Pol η) (residues 1–432) was cloned into a modified pET28p vector with a N-terminal 6-histidine tag and a PreScission Protease cleavage site as described (37). For protein expression, this Pol η plasmid was transformed into BL21 DE3 *E. coli* cells. When the optical density of the *E. coli* cells reached 0.8, isopropyl ß-D-1-thiogalactopyranoside (IPTG) was added to a final concentration of 1 μM IPTG. After 20 h (16 °C) of induction, the cell paste was collected *via* centrifugation and re-suspended in a buffer that contained 20 mM Tris (pH 7.5), 1 M NaCl, 20 mM imidazole, and 5 mM ß-mercaptoethanol (BME). After sonification, Pol η was loaded onto a HisTrap HP column (GE Healthcare), which was pre-equilibrated with a buffer that contained 20 mM Tris (pH 7.5), 1 M NaCl, 20 mM imidazole, and 5 mM BME. The column was washed with 300 ml of buffer to remove non-specific bound proteins and was eluted with a buffer that contained 20 mM Tris (pH 7.5), 1 M NaCl, 300 mM imidazole, and 3 mM dithiothreitol (DTT). The eluted Pol η was incubated with PreScission Protease to cleave the N-terminal 6-histidine-tag. Afterwards, Pol η was buffer-exchanged and desalted to 20 mM 2-(N-morpholino)ethanesulfonic acid (MES) (pH 6.0), 250 mM KCl, 10% glycerol, 0.1 mM ethylenediaminetetraacetic acid (EDTA), and 3 mM DTT and was loaded onto a MonoS 10/100 column (GE Healthcare). The protein was eluted with an increasing salt (KCl) gradient. Finally, Pol η was cleaned with a Superdex 200 10/300 Gl column (GE Healthcare) with a buffer that contained 20 mM Tris (pH 7.5), 450 mM KCl, and 3 mM DTT.

### Synthesizing araC- and gemC-terminated DNA primers

We employed an enzymatic assay to prepare araC- or gemC-terminated DNA primers. The reaction mixture contained 35 to 400 nM RB69 polymerase, 1 to 65 μM DNA, 100 μM araCTP (Jenabioscience) or gemCTP (Jenabioscience), 100 mM KCl, 50 mM Tris (pH 7.5), 5 mM MgCl_2_, 3 mM DTT, 0.1 mg/ml bovine serum albumin, and 4% glycerol. The DNA substrates were comprised of primers (5′- GTG CCT AGC GTA A -3′, fluorescein-labeled 5′- GTG CCT AGC GTA A -3′, or 5′- AGC GTC A -3′) templated to their respective complementary templates (5′- GAG TCA CTG T TAC GCT AGG CAC-3′, 5′- GAG TCA CTG T TAC GCT AGG CAC-3′, or 5′- AGG CAC CAT TGT GAC GCT -3′). Reactions were conducted at 37 °C for 1 h and were stopped by adding formamide quench buffer to the final concentrations of 40% formamide, 50 mM EDTA (pH 8.0), 0.1 mg/ml xylene cyanol, and 0.1 mg/ml bromophenol. After heating to 97 °C for 5 min and immediately placing on ice, reaction products were resolved on 22.5% polyacrylamide urea gels. Subsequently, the product bands were excised and desalted and later used in biochemical assays and crystallization.

### Cytidine analogue incorporation assay

The nucleotide incorporation activity was assayed by the following: The reaction mixture contained 1.3 to 180 nM WT Pol η, 5 μM DNA, 0 to 400 μM dCTP, araCTP (Jenabioscience), or gemCTP (Jenabioscience), 100 mM KCl, 50 mM Tris (pH 7.5), 5 mM MgCl_2,_ 3 mM DTT, 0.1 mg/ml bovine serum albumin, and 4% glycerol. The incorporation assays were executed using DNA template (5′- GAG TCA CTG T TAC GCT AGG CAC-3′) and 5′-fluorescein- labelled primer (5′- GTG CCT AGC GTA A -3′). Reactions were conducted at 37 °C for 5 min and were stopped by adding formamide quench buffer to the final concentrations of 40% formamide, 50 mM EDTA (pH 8.0), 0.1 mg/ml xylene cyanol, and 0.1 mg/ml bromophenol. After heating to 97 °C for 5 min and immediately placing on ice, reaction products were resolved on 22.5% polyacrylamide urea gels. The gels were visualized by a Sapphire Biomolecular Imager and quantified using the built-in software. Quantification of K_cat_, K_M_, V_Max_ and fitting and graphic representation were executed by Graph Prism. Source data of urea gels are provided as a Source Data file.

### DNA extension assay

The nucleotide incorporation activity was assayed by the following: The reaction mixture contained 1.3 to 180 nM WT Pol η, 500 nM DNA, 0 to 400 μM dATP, 100 mM KCl, 50 mM Tris (pH 7.5), 5 mM MgCl_2,_ 3 mM DTT, 0.1 mg/ml bovine serum albumin, and 4% glycerol. The incorporation assays were executed using DNA template (5′- GAG TCA CTG T TAC GCT AGG CAC-3′) and 5′-fluorescein- labeled primer (5′- GTG CCT AGC GTA AX -3′) with X=dC, araC, or gemC. Reactions were conducted at 37 °C for 5 min and were stopped by adding formamide quench buffer to the final concentrations of 40% formamide, 50 mM EDTA (pH 8.0), 0.1 mg/ml xylene cyanol, and 0.1 mg/ml bromophenol. After heating to 97 °C for 5 min and immediately placing on ice, reaction products were resolved on 22.5% polyacrylamide urea gels. The gels were visualized by a Sapphire Biomolecular Imager and quantified using the built-in software. Quantification of K_cat_, K_M_, V_Max,_ and fitting and graphic representation were executed by Graph Prism. Source data of urea gels are provided as a Source Data file.

### Crystallization

Pol η was concentrated to 300 μM in a buffer that contained 20 mM Tris (pH 7.5), 0.45 M KCl, and 3 mM DTT. Then DNA, dCTP, araCTP, or gemCTP, and Ca^2+^ and low salt buffer (20 mM Tris (pH 7.5), and 3 mM DTT) were added to this polymerase solution at the molar ratio of 1 : 1.2: 1: one for Pol η, DNA, dCTP, araCTP, or gemCTP, and Ca^2+^, bringing Pol η′s concentration to 100 μM. Then after this solution was kept on ice for 10 min, more dCTP, araCTP, or gemCTP was added to a final concentration of 0.5 mM. The DNA template and primer used for crystallization were 5′-CAT GAT GAC GCT-3′ and 5′-AGC GTC AT-3, respectively.

To 300 μM Pol η, araC-terminated DNA, dAMPNPP, and Mg^2+^ and low salt buffer (20 mM Tris (pH 7.5), and 3 mM DTT) were added to this polymerase solution at the molar ratio of 1 : 1.2: 1: one for Pol η, araC-terminated DNA, dAMPNPP, and Mg^2+^, bringing Pol η′s concentration to 100 μM. Then after this solution was kept on ice for 10 min, more dAMPNPP was added to a final concentration of 0.5 mM. DNA template and primer used for crystallization were 5′-CAT GAT GAC GCT-3′ and 5′-AGC GTC AaraC-3, respectively.

To crystalize Pol η with gemC at the primer terminus, DNA template and primer 5′-CAT GAT GAC GCT-3′ and 5′-AGC GTC A-3′, respectively were used. To 300 μM Pol η, DNA, gemCTP, and Mg^2+^ and low salt buffer (20 mM Tris (pH 7.5), and 3 mM DTT) were added to this polymerase solution at the molar ratio of 1 : 1.2: 1: 1 for Pol η, DNA, gemCTP, and Mg^2+^, bringing Pol η′s concentration to 100 μM. Then this solution was placed at room temperature for 10 min and then subsequentially on ice for 10 min. Afterwards, dAMPNPP and Mg^2+^ were added to a final concentration of 2 mM and 5 mM respectively.

All crystals were obtained using the hanging-drop vapor-diffusion method against a reservoir solution containing 0.1 M MES (pH 6.0) and 9 to 15% (w/v) PEG2K-MME at room temperature within 4 days.

### Chemical reaction *in crystallo*

The crystals of Pol η, araC-terminated DNA, dATP, and Ca^2+^ were first transferred and incubated in a pre-reaction buffer containing 0.1 M MES (pH 7.0, titrated by KOH), 100 μM dATP, and 20% (w/v) PEG2K-MME for 30 min. The chemical reaction was initiated by transferring the crystals into a reaction buffer containing 0.1 M MES (pH 7.0), 20% (w/v) PEG2K-MME, and 1 to 20 mM MgCl_2_ or MnCl_2_. After incubation for a desired time period, the crystals were quickly dipped in a cryo-solution supplemented with 20% (w/v) glycerol and flash-cooled in liquid nitrogen.

### Data collection and refinement

Diffraction data were collected at 100 K on LS-CAT beam lines 21-D-D, 21-ID-F, and 21-ID-G at the Advanced Photon Source (Argonne National Laboratory) or beamlines 5.0.3. at ALS. Data were indexed in space group P6_1_, scaled, and reduced using XDS (65). Isomorphous Pol η structures with Mg^2+^ PDB: were used as initial models for refinement using PHENIX (66) and COOT (67). Occupancies were assigned for the substrate, reaction product, PP_i_, Me^2+^_A_, and Me^2+^_B_ for the ternary ground state, following the previous protocol (34) until there were no significant F_o_-F_c_ peaks and each atom’s B value had roughly similar values to its ligand. Occupancies were assigned to the misaligned and aligned conformations of the primer termini until there were no more significant F_o_-F_c_ peaks. For the structures in which both primer conformations were at an equilibrium (not 100% in either misaligned or aligned), occupancies were assigned until the F_o_-F_c_ peaks for both conformations (while they still remained) did not increase (41). In addition, for the structures in which some positive F_o_-F_c_ peaks were present around the Me^2+^ binding sites or primer termini, no change in the assigned occupancy was executed when a 10% change in occupancy (*e.g.* 100 to 90%) failed to significantly change the intensity of the F_o_-F_c_ peaks. Source data of the electron densities in r.m.s. density are provided as a Source Data file. Each structure was refined to the highest resolution data collected, which ranged between 1.52 to 2.2 Å. Software applications used in this project were compiled and configured by SBGrid (68). Source data of data collection and refinement statistics are summarized in Table S2, *A*–*C*. All structural figures were drawn using PyMOL (http://www.pymol.org).

## Data availability

The coordinates, density maps, and structure factors for all the structures have been deposited in Protein Data Bank (PDB) under accession codes: 8V7A, 8V7B, 8V7C, 8V7D, 8V7E, 8V7F, 8V7G, 8V7H, 8V7I, 8V7J, and 8V7K.

## Supporting information

This article contains supporting information.

## Conflict of interest

The authors declare that they have no conflicts of interest with the contents of this article.
