# Supplementary material for: Sugar ring alignment and dynamics underline cytarabine and gemcitabine inhibition on Pol η catalyzed DNA synthesis
Source: J Biol Chem. 2024 May 10;300(6):107361. doi: 10.1016/j.jbc.2024.107361 (PMC11176770; doi:10.1016/j.jbc.2024.107361)
Supplement: Supporting Information [file mmc1.pdf]

Supporting Information

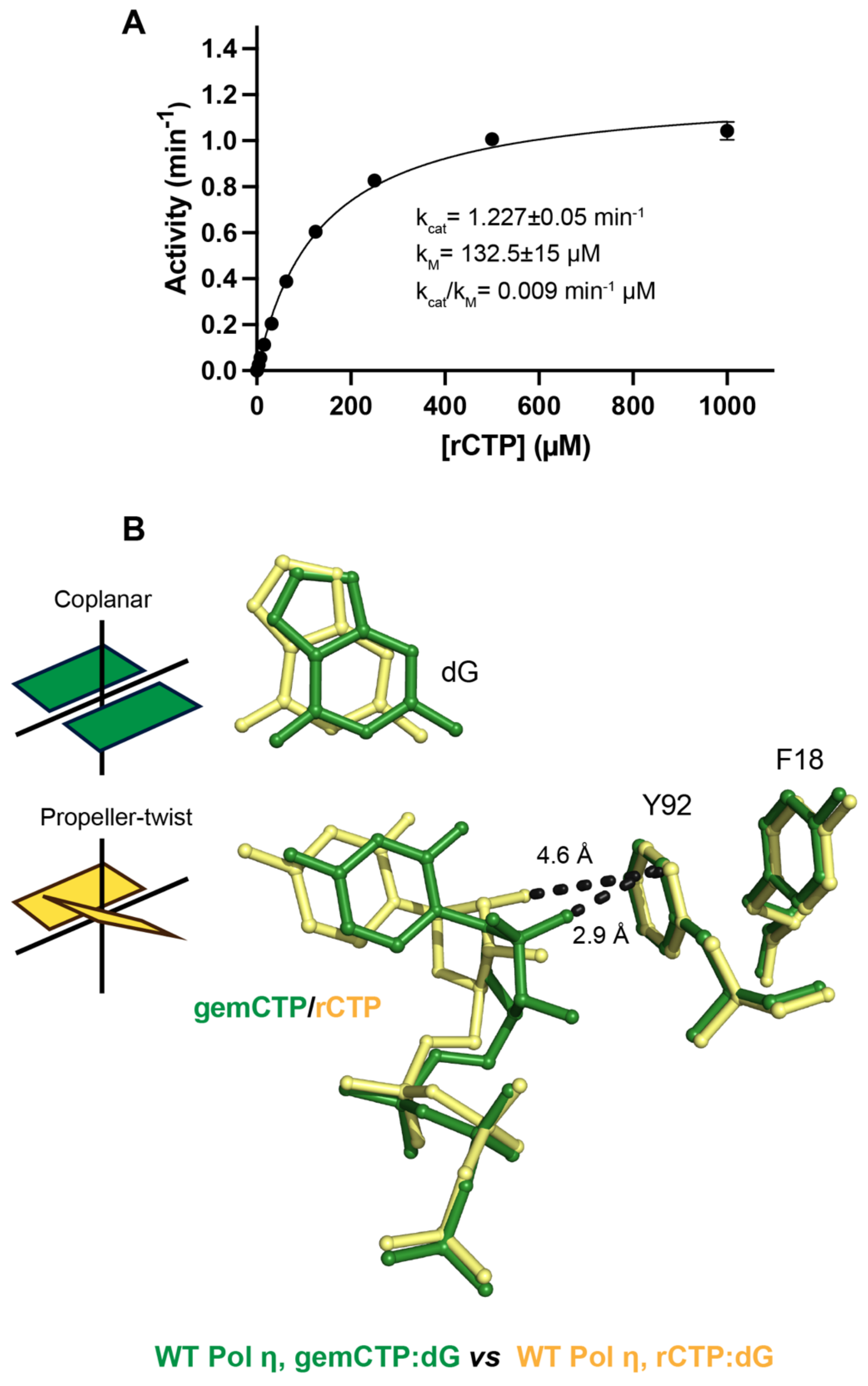

Supplementary Figure 1 Biochemical and structural characterizations of rCTP incorporation by Pol η.

A, steady-state kinetics of Pol η incorporating rCTP**.** Each data point represents the mean of triplicate measurements for the polymerase incorporation rate while the errors bars represent the standard deviation. *B*, structural overlay of gemCTP (green) and rCTP

(yellow) during the incorporation step. The distances between the 2´-modifications on the nucleotide and the steric gate are indicated.

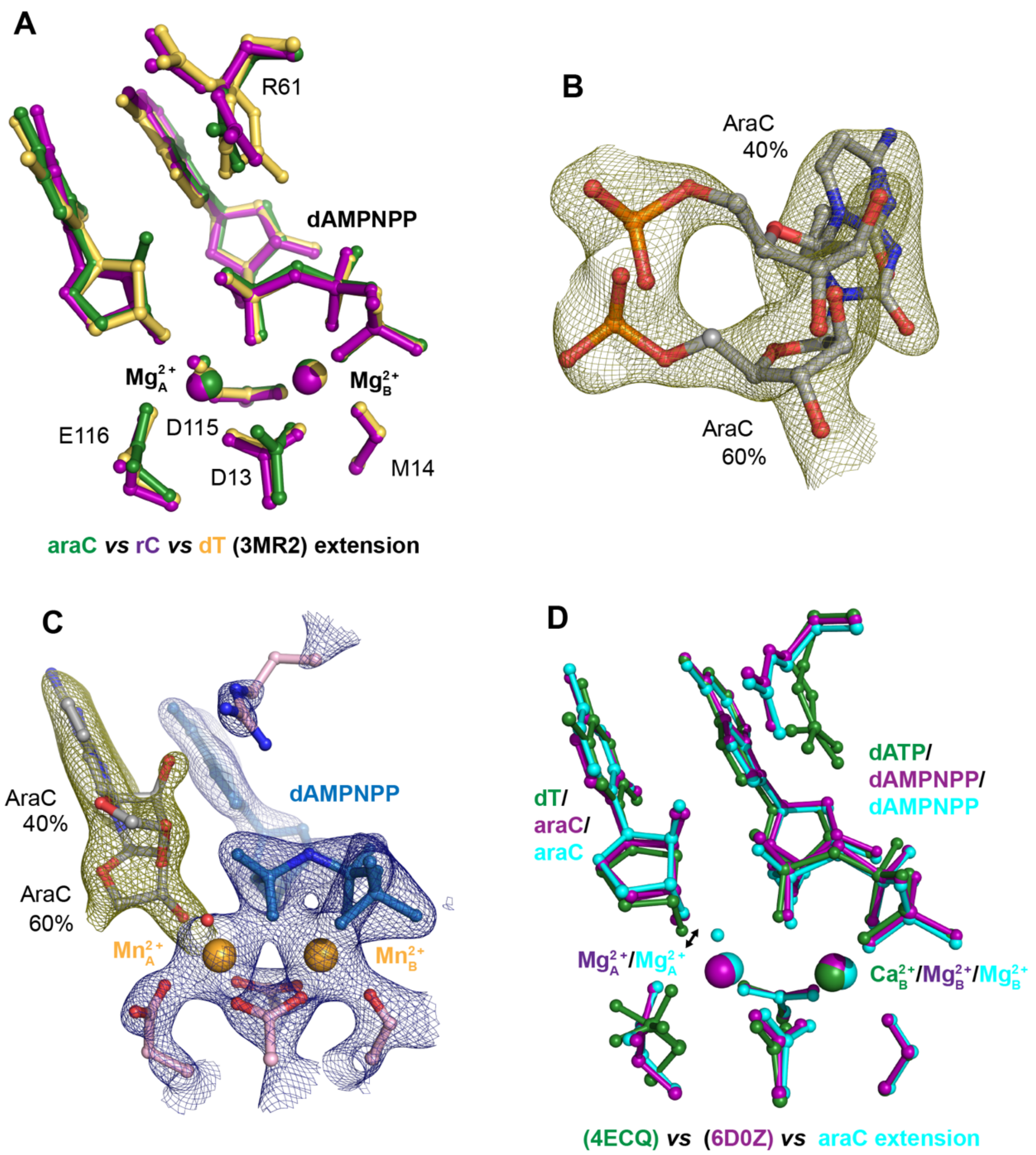

Supplementary Figure 2 Pol η active site during araC extension bound with different Me^{2+} and nucleotide analogues**.**

A, structural overlay of the down conformation of the primer terminus during araC extension (green), rC extension (purple) and dT extension (PDB ID **3MR2**) (yellow). The conformational changes of the Arg61 sidechain and the sugar pucker at the primer terminus are evident. *B*, active site conformations of the primer terminus during araC extension. *C*, active site structure of araC extension complex with Mn^{2+}. *D*, structural overlay of dT extension structure with dATP as the incoming nucleotide (green), published araC extension (PDB ID **6D0Z**) (purple) and our araC extension (cyan) structures with dAMPNPP as the incoming nucleotide. *B, C*, the $2F_o$-F_c map for $Me^{2+}{}_A$ and $Me^{2+}{}_B$,

dAMPNPP, and catalytic residues (blue) was contoured at 2 σ. The F_o-F_c omit map for the primer terminus (green) was contoured at 2.5 σ.

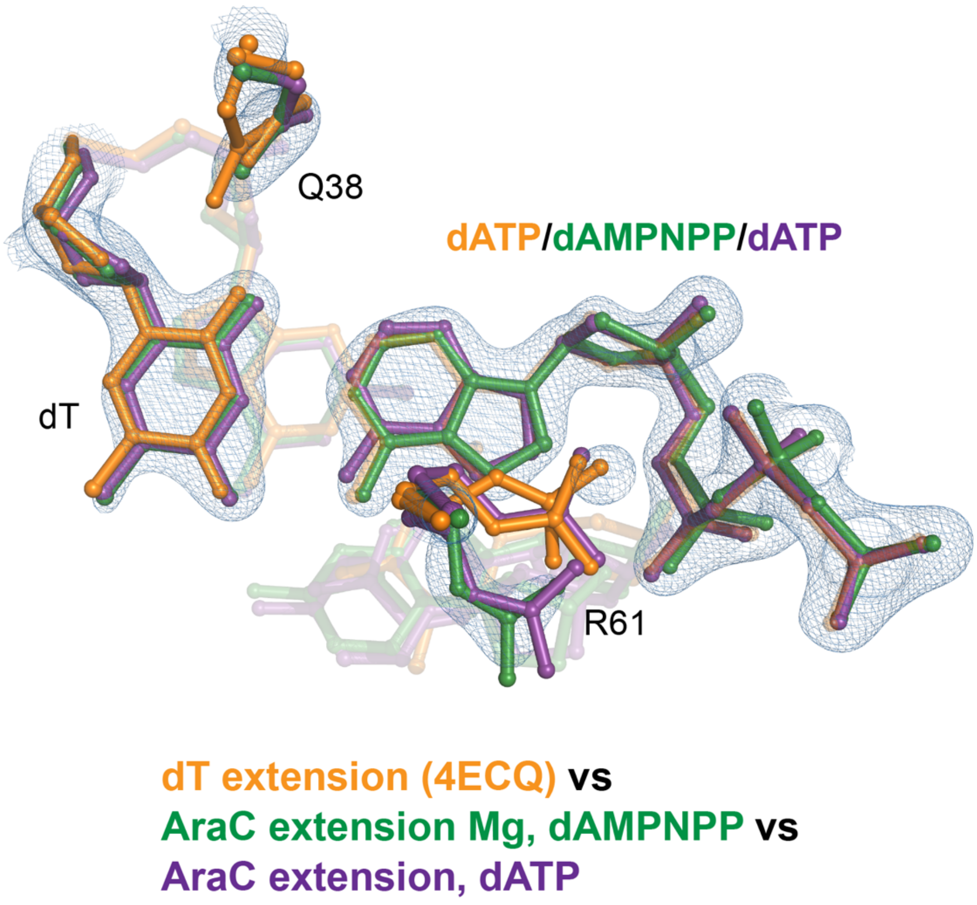

Supplementary Figure 3 Nucleotide basepair geometry during araC extension by Pol η.

Structure overlay of Pol η complexes with incoming dATP, Ca^{2+} and dT at the primer terminus (PDB ID 4ECQ, yelloworange), incoming dAMPNPP, Mg^{2+}, and araC at the primer terminus (green), and incoming dATP, Ca^{2+}, and araC at the primer terminus (purple). The alignment indicated a difference in the R61 sidechain conformation but minimal changes in the basepairing geometries. The 2F_o-F_c map for the dT template base, incoming nucleotide, and catalytic residues (blue) was contoured at 1.8 σ and applies to the structure colored in green.

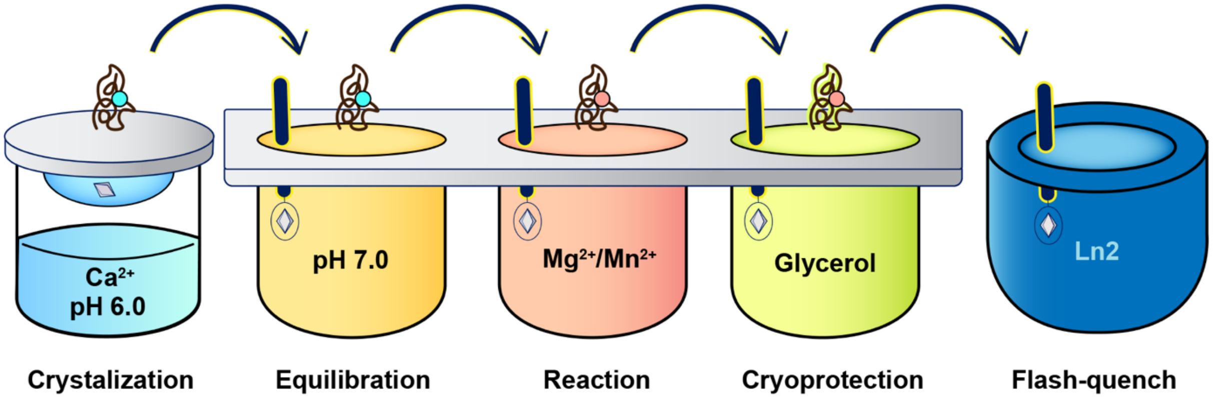

Supplementary Figure 4 In crystallo setup for visualizing araC extension by Pol η. The crystal of the ternary complex of Pol η (Pol η with araC terminated DNA) is grown with Ca^{2+} at pH 6.0 in the hanging drop. Pol η crystals were looped out from the hanging drop after ~4 days of crystal growth and then first soaked in the equilibration buffer (crystallization condition with a pH of 7.0) to change the pH to pH 7.0. Afterwards, the *in crystallo* reaction was initiated by looping and soaking the crystal in the reaction buffer (equilibration buffer plus Mg^{2+} or Mn^{2+}). After soaking for a defined period of time, the crystal was looped and soaked in the cryoprotection buffer (reaction buffer plus 20% glycerol) for 1 second. Finally, the crystal was rapidly immersed in liquid nitrogen and stored for X-ray analysis.

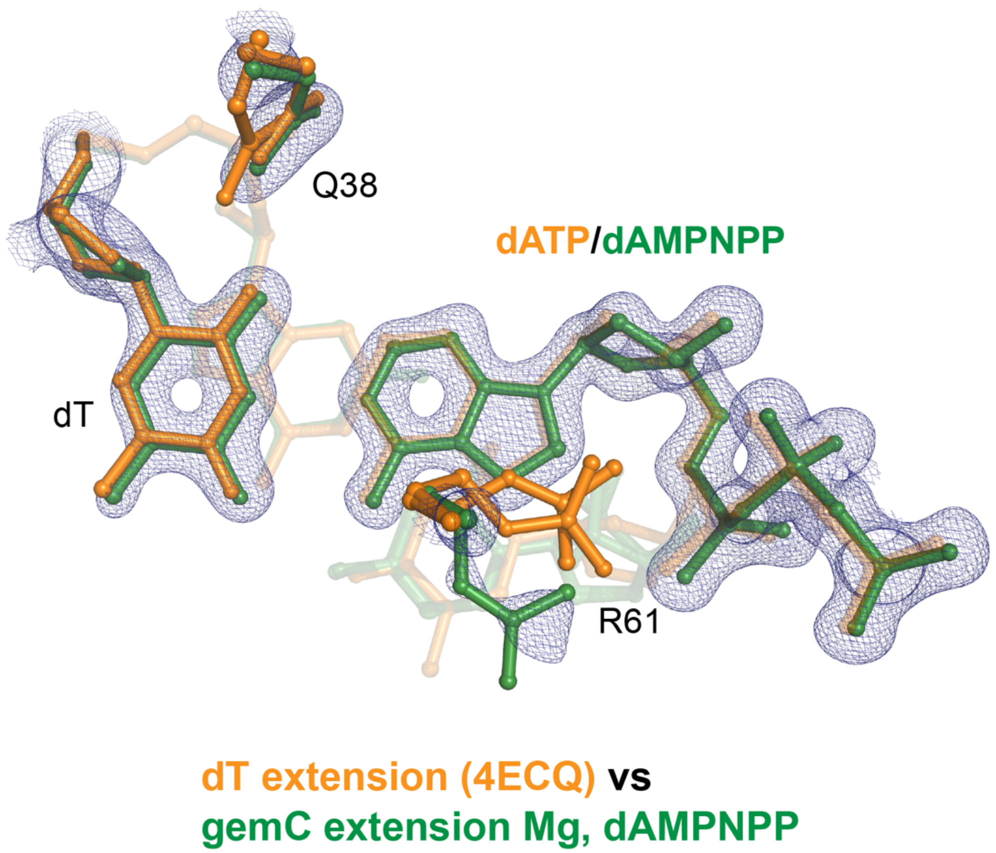

Supplementary Figure 5 Nucleotide basepair geometry during gemC extension by Pol η.
Structure overlay of Pol η complexes with ATP, Ca^{2+} and dT at the primer terminus (PDB ID 4ECQ, yelloworange) versus with incoming dAMPNPP, Mg^{2+}, and gemC at the primer terminus gemC (green). The alignment indicated a difference in the R61 sidechain conformation but minimal changes in the basepairing geometries.The $2F_o$-F_c map for the dT template base, incoming nucleotide, and catalytic residues (blue) was contoured at 1.8 σ and applies to the structure colored in green.

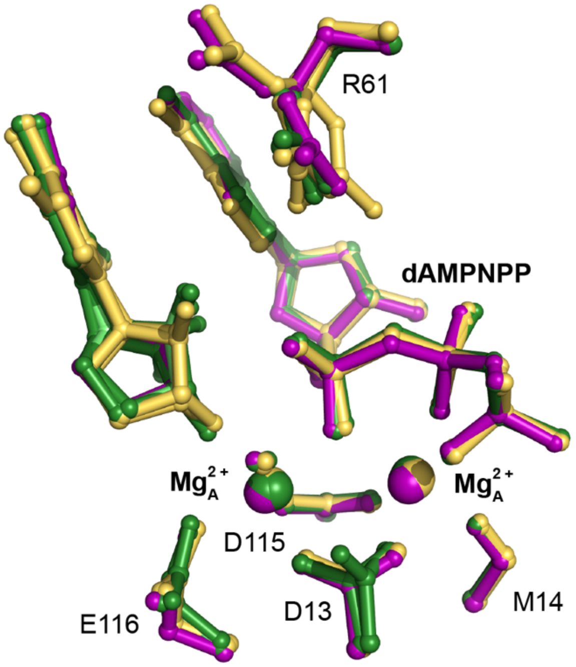

Supplementary Figure 6 Pol η active site during gemC extension.
Structural overlay of the Pol η active site during gemC extension (green), rC extension (purple) and dT extension (PDB ID **3MR2**) (yellow) with dAMPNPP as the incoming nucleotide. The alignment indicated changes in the Arg61 sidechain and sugar pucker at the primer terminus.

Supplementary Table 1: Crystal Diffraction and refinement data.

A Pol η insertion ternary complex

	dCTP	araCTP	gemCTP	rCTP
**PDB Code**	8V7A	8V7B	8V7C	8V7D
**Data collection**				
Wavelength (Å)	0.9786	0.9786	0.9787	0.9786
Space group	$P6_1$	$P6_1$	$P6_1$	$P6_1$
Cell dimensions				
a, b, c (Å)	98.193<br>98.193<br>81.746	98.848<br>98.848<br>82.048	98.85<br>98.85<br>82.12	99.058<br>99.058<br>81.386
α, β, γ (˚)	90, 90, 120	90, 90, 120	90, 90, 120	90, 90, 120
Resolution (Å)[1]	42.52 - 1.95<br>(2.02 - 1.95)	42.34 - 1.9<br>(1.97 - 1.9)	42.81 - 1.79<br>(1.85 - 1.79)	42.31 - 1.95<br>(2.02 - 1.95)
R_{sym} or R_{merge}[1]	0.0622 (0.8541)	0.0753 (1.274)	0.0679 (0.8371)	0.0681 (1.019)
$I/\sigma I$[1]	24.48 (2.90)	23.11 (1.96)	21.63 (3.06)	24.70 (2.60)
$CC^{1/2}$[1]	1 (0.837)	1 (0.726)	0.999 (0.877)	1 (0.789)
Completeness (%)	99.98 (100.00)	99.98 (100.00)	99.99 (100.00)	99.98 (100.00)
No. unique reflections[1]	32717<br>(3263)	35938<br>(3565)	42984<br>(4271)	33142<br>(3283)
**Refinement**				
Nucleotide occ.	1.00	1.00	0.85	1.00
A site occupancy	0.35 K^+	0.30 K^+	-	0.30 K^+
B site occupancy	1.00 Ca^{2+}	1.00 Ca^{2+}	0.85 Ca^{2+}	1.00 Ca^{2+}
B-factors				
Protein	38.02	34.92	32.3	37.35
DNA	42.13	38.57	35.59	40.68
Ligand	26.65	25.3	29.28	32.39
Water	45.89	40.46	38.49	45.23
Resolution (Å)	1.95	1.90	1.79	1.95
No. reflections	32716 (3263)	35935 (3565)	42984 (4271)	33140 (3283)
R_{work}/R_{free}	0.17/0.22	0.18/0.21	0.18/0.21	0.18/0.23
Wilson B	32.98	31.15	26.64	32.97
Ramachandran				
Favored (%)	97.18	96.71	97.18	98.12
Outlier (%)	1.04	0.79	1.57	0.79
R.m.s. deviations				
Bond lengths (Å)	0.009	0.008	0.008	0.009
Bond angles (˚)	1.1	1.05	1.16	1.15

[1]Data in the highest resolution shell is shown in the parenthesis.

B Pol η extension ternary complex

	AraC-Mg^{2+}	AraC-Mn^{2+}	GemC-Mg^{2+}
**PDB Code**	8V7E	8V7F	8V7G
**Data collection**			
Wavelength (Å)	0.9786	0.9786	0.97648
Space group	*P6₁*	*P6₁*	
Cell dimensions			
a, b, c (Å)	98.759	98.7	98.36
	98.759	98.7	98.36
	81.97	82.324	82.05
α, β, γ (˚)	90, 90, 120	90, 90, 120	90, 90, 120
Resolution (Å)[1]	42.3 - 1.82	42.74 - 2.2	42.18 - 1.52
	(1.89 - 1.82)	(2.28 - 2.2)	(1.57 - 1.52)
R_{sym} or R_{merge}[1]	0.052 (0.8103)	0.090 (0.7437)	0.039 (0.6408)
$I/\sigma I$[1]	27.41 (2.84)	22.21 (3.49)	34.03 (2.90)
$CC^{1/2}$ [1]	1 (0.867)	0.997 (0.875)	1 (0.844)
Completeness (%)	99.99 (100.00)	99.84 (99.83)	99.98 (99.88)
No. unique	40758	23193	69275
reflections[1]	(4045)	(2306)	(6882)
**Refinement**			
dAMPNPP occ.	1.00	1.00	1.00
Misaligned primer occ.	0.40	0.40	0.30 C2´-endo
Non-misaligned primer occ.	0.60	0.60	0.70 C3´-endo
A site occupancy	0.50 Mg^{2+}	1.00 Mn^{2+}	1.00 Mg^{2+}
B site occupancy	1.00 Mg^{2+}	1.00 Mn^{2+}	1.00 Mg^{2+}
B-factors			
Protein	33.82	38.04	24.91
DNA	37.21	42.65	28.05
Ligand	26.33	26.91	15.83
Water	38.51	30.79	32.7
Resolution (Å)	1.82	2.20	1.52
No. reflections	40757 (4045)	23188 (2306)	69274 (6882)
R_{work}/R_{free}	0.17/0.21	0.20/0.25	0.18/0.20
Wilson B	29.13	33.2	18.41
Ramachandran			
Favored (%)	97.89	98.33	97.42
Outlier (%)	0.7	0.24	0
R.m.s. deviations			
Bond lengths (Å)	0.009	0.004	0.008
Bond angles (˚)	1	0.7	1.06

[1]Data in the highest resolution shell is shown in the parenthesis.

C Pol η Mg^{2+}/Mn^{2+} *in crystallo* soaking

	AraC ground	1 mM Mg^{2+} 1800s	20 mM Mg^{2+} 600s	10 mM Mn^{2+} 1800s
**PDB Code**	8V7H	8V7I	8V7J	8V7K
**Data collection**				
Wavelength (Å)	1.127	0.9786	0.9786	1.127
Space group	$P6_1$	$P6_1$	$P6_1$	$P6_1$
Cell dimensions				
a, b, c (Å)	98.4<br>98.4<br>81.57	98.54<br>98.54<br>81.89	98.72<br>98.72<br>82.09	98.52<br>98.52<br>81.65
α, β, γ (˚)	90, 90, 120	90, 90, 120	90, 90, 120	90, 90, 120
Resolution (Å)[1]	37.77 - 1.68 (1.74 - 1.68)	32.26 - 1.72 (1.78 - 1.72)	37.92 - 1.66 (1.72 - 1.66)	32.25 - 1.66 (1.71 - 1.66)
R_{sym} or R_{merge}[1]	0.0473 (0.953)	0.0585 (0.816)	0.063 (0.847)	0.0667 (1.006)
$I/\sigma I$[1]	35.49 (2.96)	28.92 (3.05)	26.44 (3.19)	27.14 (2.71)
$CC^{1/2}$ [1]	1 (0.895)	0.999 (0.861)	1 (0.849)	1 (0.865)
Completeness (%)	99.95 (99.68)	99.02 (98.09)	99.79 (98.21)	99.91 (99.25)
No. unique reflections[1]	50730 (5017)	47526 (4679)	53145 (5210)	53652 (5306)
**Refinement**				
dATP occ.	0.75	0.80	0.80	0.80
Misaligned primer occ.	0.50	0.70	0.70	0.30
Non-misaligned primer occ.	0.50	0.30	0.30	0.70
A site occupancy	0.20 K^+	0.40 Mg^{2+}	0.70 Mg^{2+}	0.70 Mn^{2+}
B site occupancy	0.75 Ca^{2+}	0.80 Mg^{2+}	0.80 Mg^{2+}	0.80 Mn^{2+}
B-factors				
Me_A/Lig_A [2]	35.3/40.97	26.7/37.1	26.3/36.5	29.3/37.5
Me_B/Lig_B [2]	35.2/36.2	23.6/31.1	20.5/26.8	28.1/30.9
Protein	34.13	26.72	26.24	31.88
DNA	38.07	31.38	31.55	37.76
Ligand	34.96	31.39	27.05	33.75
Water	31.39	23.53	25.73	30.09
Resolution (Å)	1.68	1.72	1.66	1.66
No. reflections	50723 (5015)	47521 (4679)	53141 (5209)	53649 (5306)
R_{work}/R_{free}	0.21/0.23	0.21/0.23	0.20/0.22	0.21/0.23
Wilson B	29.57	22.2	20.18	25.24
Ramachandran				
Favored (%)	97.85	97.85	97.85	97.85
Outlier (%)	0.24	0.48	0	0.24
R.m.s. deviations				
Bond lengths (Å)	0.008	0.01	0.009	0.012
Bond angles (˚)	1.14	1.24	1.18	1.32

[1]Data in the highest resolution shell is shown in the parenthesis.
[2]B-factor of metal ions and their protein nucleotide ligands.
